# Obligatory Role of EP1 Receptors in the Increase in Cerebral Blood Flow Produced by Hypercapnia in the Mice

**DOI:** 10.1371/journal.pone.0163329

**Published:** 2016-09-22

**Authors:** Ken Uekawa, Kenzo Koizumi, Jason Hwang, Nathalie Brunier, Yorito Hattori, Ping Zhou, Laibaik Park

**Affiliations:** Feil Family Brain and Mind Research Institute, Weill Cornell Medical College, New York, NY, 10065, United States of America; University of North Carolina at Chapel Hill, UNITED STATES

## Abstract

Hypercapnia induces potent vasodilation in the cerebral circulation. Although it has long been known that prostanoids participate in the cerebrovascular effects of hypercapnia, the role of prostaglandin E2 (PGE_2_) and PGE_2_ receptors have not been fully investigated. In this study, we sought to determine whether cyclooxygenase-1 (COX-1)-derived PGE_2_ and EP1 receptors are involved in the cerebrovascular response induced by hypercapnia. Cerebral blood flow (CBF) was recorded by laser-Doppler flowmetry in the somatosenasory cortex of anesthetized male EP1^-/-^ mice and wild type (WT) littermates. In WT mice, neocortical application of the EP1 receptor antagonist SC-51089 attenuated the increase in CBF elicited by systemic hypercapnia (pCO_2_ = 50–60 mmHg). SC-51089 also attenuated the increase in CBF produced by neocortical treatment of arachidonic acid or PGE_2_. These CBF responses were also attenuated in EP1^-/-^ mice. In WT mice, the COX-1 inhibitor SC-560, but not the COX-2 inhibitor NS-398, attenuated the hypercapnic CBF increase. Neocortical application of exogenous PGE_2_ restored the attenuation in resting CBF and the hypercapnic response induced by SC-560. In contrast, exogenous PGE_2_ failed to rescue the attenuation both in WT mice induced by SC-51089 and EP1^-/-^ mice, attesting to the obligatory role of EP1 receptors in the response. These findings indicate that the hypercapnic vasodilatation depends on COX-1-derived PGE_2_ acting on EP1 receptors and highlight the critical role that COX-1-derived PGE_2_ and EP1 receptors play in the hypercapnic regulation of the cerebral circulation.

## Introduction

Cerebral blood vessels are highly sensitive to changes in arterial partial pressure of carbon dioxide (pCO_2_). Hypercapnia is a potent vasodilator in the cerebrovascular microcirculation [1]. Even though many factors are likely involved in vasodilatation induced by hypercapnia [2–4], evidence suggests that prostanoids synthesized by cyclooxygenase (COX)-1 or -2 from arachidonic acid (AA) play a critical role in the hypercapnic response of the cerebral microcirculation. For example, inhibition of COX by indomethacin, an agent that inhibits both COX-1 and COX-2, attenuates the increase in CBF induced by hypercapnia [1, 5]. More recently, using the highly selective COX-1 inhibitor SC-560 and COX-1-null mice it was found that COX-1 is required for the cerebrovascular regulation induced by hypercapnia [6]. However, the pathway downstream of COX-1 driving vasodilation in response to hypercapnia is not well understood.

Recent findings suggest that prostaglandin E2 (PGE_2_), the major prostanoid formed in microvessels [7], is implicated in the cerebrovascular regulation [8–10]. PGE_2_ exerts its effect by acting on 4 G-protein coupled membrane receptors (EP1 through EP-4) [11]. Depending on the receptors PGE_2_ acts on, it can induce either vasoconstriction or vasodilation. For example, when PGE_2_ acts on EP1 receptors in perivascular nerves, it induces vasodilation [12]. Furthermore, EP1 receptors in cerebral endothelial cells have been implicated in the cerebrovascular effects of hypertension [13, 14]. However, it is unknown whether PGE_2_ acting on EP1 receptors play a role in the regulation of cerebrovascular response evoked by hypercapnia.

In the present study, we sought to determine whether COX-1-derived PGE_2_ and EP1 receptors are involved in the cerebrovascular response induced by hypercapnia. Using pharmacological probes and EP1 null mice, we found that the hypercapnic vasodilatation in the somatosensory cortex depends on COX-1-derived PGE_2_ acting on EP1 receptors. Therefore, the findings highlight the critical role that COX-1 derived prostanoids and EP1 receptors play in the regulation of the cerebral circulation.

## Materials and Methods

### 1. Mice

All experimental procedures were approved by the Institutional Animal Care and Use Committee of Weill Cornell Medical College. Experiments were performed in 3–4 months old male mice. PGE_2_ EP1-deficient mice were obtained from our in-house colony and were congenic with the C57BL/6 strain [15]. C57BL/6 mice were obtained from the Jackson Laboratory and used for wild types control.

### 2. General surgical procedures

Procedures for surgical preparation of the mice have been described previously in detail [16–18] and are only summarized here. Mice were anesthetized with isoflurane (induction: 5%; maintenance: 1–2%). One of the femoral arteries was randomly selected and cannulated for recording of arterial pressure and collection of blood samples. Mice were intubated and artificially ventilated with an oxygen-nitrogen mixture adjusted to provide an arterial pO_2_ (pO_2_) of 120–140 mmHg (S1–S7 Tables). Rectal temperature was maintained at 37°C using a thermostatically controlled rectal probe connected to a heating pad. After surgery, isoflurane was gradually discontinued and anesthesia was maintained with urethane (750 mg/kg; i.p.) and α-chloralose (50 mg/kg; i.p.). Throughout the experiment, the level of anesthesia was monitored by testing corneal reflexes and motor responses to tail pinch.

### 3. Monitoring of cerebral blood flow

A small craniotomy (2x2 mm) was performed to expose the somatosensory cortex. The dura was removed, and the site was superfused with a modified Ringer’s solution (37° C; pH: 7.3–7.4)(see [19] for composition). CBF was continuously monitored at the site of superfusion with a laser-Doppler probe (Vasamedic, St. Paul, MN) positioned stereotaxically on the neocortical surface and connected to a computerized data acquisition system. Resting CBF values were recorded by the laser-Doppler probe and presented as raw data in laser-Doppler perfusion units (LDU). CBF values were expressed as percent increase relative to the resting level. Zero values for CBF were obtained after the heart was stopped by an overdose of isoflurane at the end of the experiment.

### 4. Immunofluorescent staining and confocal microscopy

Anesthetized mice were perfused transcardially with heperinized saline, followed by 4% (wt/vol) paraformaldehyde [17, 18]. Brains were taken, postfixed overnight in the same fixative, and cut in 40 μm thickness. Brain sections were randomly selected and processed for labeling COX-1 (1:100; Cayman Chemicals) or EP1 (1:100; Cayman Chemicals) with endothelial cells (anti-platelet endothelial adhesion molecule-1 (anti-PECAM-1 or anti-CD31, 1:50; BD Biosciences), neurons (anti-neuronal nuclei, anti-Neun; Chemicon International), astrocytes (anti-GFAP, 1:1000; Sigma), or microglia (anti-ionized calcium-binding adaptor moecule 1, anti-Iba1; 1:500; Wako Chemicals). Then, sections were incubated with a FITC or a Cy5-conjugated secondary antibody (1:200, Jackson ImmunoResearch Laboratories) and mounted on slides. The specificity of the labeling was established by omitting the primary antibody or by preabsorption with the antigen. Images were obtained using a confocal laser scanning microscope (Leica SP5) in somatosensory cortex underlying the cranial window (0.38 to −1.94 mm from Bregma). Brain sections from EP1^-/-^ mice and WT littermates were processed under identical conditions and imaged using identical settings. CD31-positve vascular profiles (the number of and the % area occupied by the profiles) and COX-1 immunoreactivity were quantified using ImageJ (National Institutes of Health).

### 5. Experimental protocol

CBF recordings were started after arterial pressure and blood gases were in a steady state (S1–S7 Tables). The cranial window was superfused with Ringer’s solution. CBF responses to whisker stimulation were recorded while gently stroking the whiskers with a cotton-tipped applicator for 60 sec. The endothelium (EC)-dependent vasodilator acetylcholine (10 μM; Sigma), endothelial nitric oxide synthase (eNOS)-dependent vasodilator [1], or A23187 (3 μM; Sigma), COX-1-dependent vasodilator [6], was topically superfused for 3–5 min and the evoked CBF increases recorded. CBF responses to adenosine (400 μM; Sigma), agents that produce vasodilation by acting directly on vascular smooth muscles [1], were also tested. The increase in CBF produced by hypercapnia was examined by introducing 5% CO_2_ in the ventilator to increase arterial pCO_2_ up to 50–60 mmHg. In the previous studies, this level of pCO_2_ was found to increase PGE_2_ release that induces vasodilation of cerebral arterioles [20–22]. Once a stable increase in CBF was obtained for 3–5 min, pCO_2_ was returned to normocapnia. All pharmacological agents were dissolved in a modified Ringer’s solution, unless otherwise written.

#### a. Dose response effect of the EP1 inhibitor SC-51089 on cerebrovascular responses induced by hypercapnia in WT and EP1-/- mice

In WT mice, after testing baseline CBF response to hypercapnia, acetylcholine, A23187, or adenosine, the superfusion solution was switched to Ringer’s containing the EP1 receptor inhibitor SC-51089 (8-chlorodibenz11[1,4]oxazepine-10(11H)-carboxylic acid; 2-[1-oxo-3-(3-pyridinyl)propyl]hydrazide (Biomol) [23]. SC-51089 was dissolved in dimethylsulfoxide (DMSO), diluted with Ringer’s to the desired concentration and superfused on the somatosensory cortex. The final DMSO concentration was <0.2% in all the experiments, which does not affect the cerebrovascular responses tested [24]. The following SC-51089 concentrations were tested: 1, 10, or 100 μM. Thirty to 40 minutes later, blood pressure and resting CBF were recorded, and CBF resposnes to hypercapnia, acetylcholine, A23187, or adenosine were examined again. In the prevous experiments, we and others established that the CBF response to hypercapnia is stable over the duration of the experiments [25–27]. In EP1^-/-^ mice, CBF response to whisker stimulation, acetylcholine, adenosine, A23187, or hypercapnia was examined with or withuot application of SC-51089. The effect of SC-51089 on CBF response produced by whisker stimulation was tested in a separate group of mice because hypercapnia affects the CBF response to whisker stimuation [25].

#### b. Effect of the EP3 inhibitor L-798,106 or the EP4 inhibitor ONO-AE3-208 on the cerebrovascular responses induced by hypercapnia in WT mice

After the baseline CBF responses were established, the superfusion solution was switched to Ringer’s containing the EP3 inhibitor L-798,106 (thiophene-2-sulphonic acid {3-[2–2-(4-methylsulphonylbenzyl)-phenyl]-acryloyl}-amide, 1 μM; Tocris) [28] or the EP4 inhibitor ONO-AE3-208 (4-{4-Cyano-2-[2-(4-fluoronaphthalen-1-yl) propionylamino] phenyl} butyric acid, 1 μM; Tocris) [29, 30]. L-798,106 or ONO-AE3-208 was dissolved in DMSO and diluted with Ringer’s. The CBF responses to hypercapnia, whisker stimulation, acetylcholine, and adenosine were tested again 30–40 minutes later (see above).

#### c. Effect of the COX-1 inhibitor SC-560 with or without PGE2 or the COX-2 inhibitor NS-398 on the cerebrovascular responses induced by hypercapnia in WT mice

After the baseline CBF responses were established, the superfusion solution was switched to Ringer’s containing the COX-1 inhibitor SC-560 ([5-(4-chlorophenyl)-1-(4-metoxyphenyl)-3-trifluoromethypyrazole], 25 μM) or the COX-2 inhibitor NS398 (N[2-(cyclohexyloxy)-4-nitrophenyl]-methanesulfonamide) (100 μM, Biomol) [6, 31]. SC-560 or NS-398 was dissolved in DMSO and diluted with Ringer’s. The CBF responses to hypercapnia (pCO_2_ = 50-60mmHg), acetylcholine, A23187, or adenosine were tested again 30–40 minutes later. The effect of SC-560 or NS-398 on CBF response produced by whisker stimulation was tested in a separate group of mice (see above).

#### d. Effect of SC-51089 on the cerebrovascular responses induced by arachidonic acid or PGE2

In these studies, the effect of a half maximal concentration of SC-51089 (10 μM) was tested on the vasodilation induced by neocortical superfusion of arachidonic acid (AA) or PGE_2_. After the baseline responses to hypercapnia, acetylcholine and adenosine were established, SC-51089 (10 μM) was superfused over the somatosensory cortex for 30–40 minutes and the CBF responses to the neocortical superfusion of different concentrations of AA (0.5, 1, 10, and 100 μM) or PGE_2_ (1, 5, 10, 20, 50, and 100 μM) were tested.

#### e. Effect of PGE2 on the attenuation of the hypercapnic vasodilation by SC-560 or SC-51089 in WT mice

The reactivity of CBF to hypercapnia (pCO_2_ = 50-60mmHg) was established first. Then, the superfusion solution was switched to Ringer’s containing SC-560 (25 μM) or SC-51089 (10 μM) and 30–40 minutes later the response to hypercapnia was tested again. Subsequently, superfusate was switched to Ringer’s containing SC-560 or SC-51089 with an sub-effective does of PGE_2_ (1 μM) (see Table 1) and the response to hypercapnia was tested again 30–40 minutes later.

**Table 1:** CBF increases induced by AA or PGE_2_.

	Dose (μM)	EP1^+/+^		EP1^-/-^	N
		Vehicle	SC-51089	Vehicle	
AA	0.5	6±1	3±1*	3±1*	5
	1	12±1	6±1*	7±1*	5
	10	20±2	9±1*	9±3*	5
	100	39±5	16±1*	19±3*	5
PGE_2_	1	1±1	1±1	1±1	5
	5	9±2	5±1*	4±1*	5
	10	16±2	10±2*	8±2*	5
	20	20±3	13±2*	14±2*	5
	50	24±4	13±2*	17±1*	5

Values (%) are means±SEM; AA, arachidonic acid; CBF, cerebral blood flow; N, number of mice; SC-51089, 10 μM

*, p<0.05 from vehicle-treated EP1^+/+^.

### 6. Data analysis

Mice were randomly assigned to treatment with inhibitors or vehicle. Data in text and figures were analyzed with Prism 7 (Version 7.0a; GraphPad Software) and are expressed as means ± SEM. Two-group comparisons were analyzed by the two-tailed t-test for dependent or independent samples, as appropriate. Multiple comparisons were evaluated by the analysis of variance (ANOVA) and Tukey’s test. When necessary, non parametric Mann Whitney test was employed to reinforce statiscal power for the sample size of N = 5/group. Statistical significance was taken for probability values of less than 0.05.

## Results

### PGE_2_ EP1 receptors are required for the increase in CBF induced by hypercapnia

**Fig 1:**
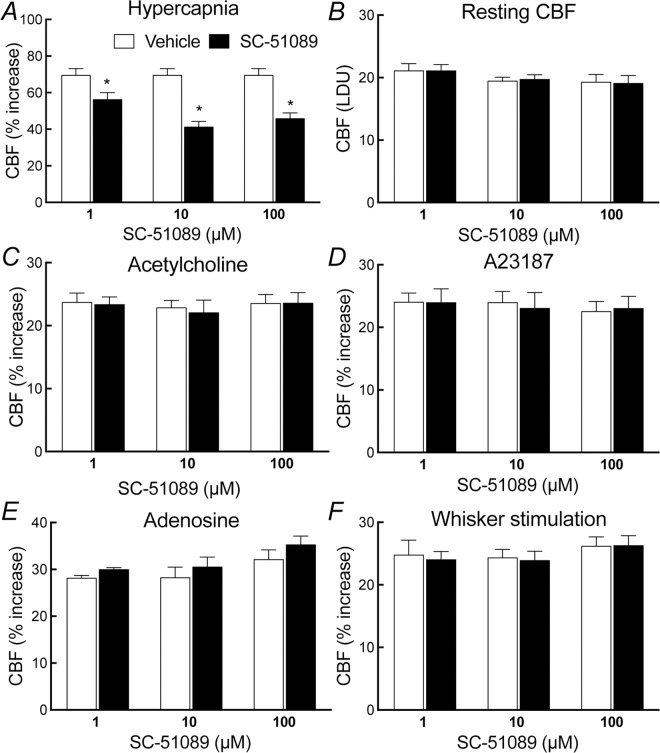
**Effect of the EP1 receptor inhibitor SC-51039 on the increase in CBF induced by hypercapnia (A), resting CBF (B), and on the increase in CBF induced by acetylcholine (C), A23187 (D), adenosine (E), or whisker stimulation (F) in wild-type mice**. LDU, laser-Doppler perfusion units; * p<0.05 from vehicle, analysis of variance and Tukey’s test; n = 5/group.

**Fig 2:**
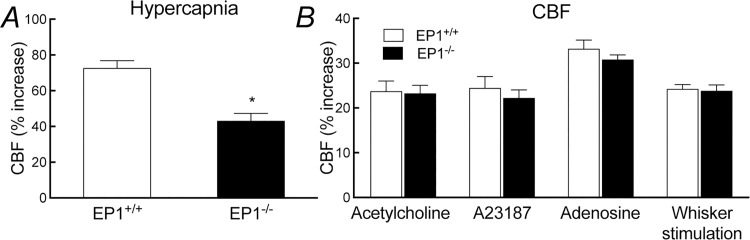
Cerebrovascular responses in EP1^-/-^ mice. A, increase in CBF produced by hypercapnia; B, CBF response produced by acetylcholine, A23187, adenosine, or whsiker stimulation. * p<0.05, paired t-test; n = 5/group.

**Fig 3:**
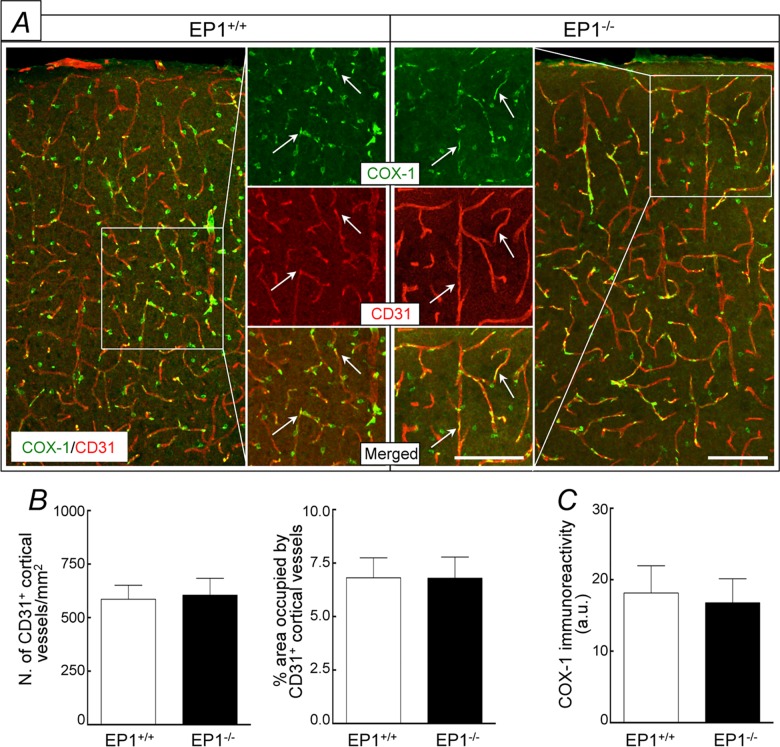
Blood vessel profiles and COX-1 expression in the somatosensory cortex of EP1^+/+^ and EP1^-/-^ mice. A, COX-1 immunoreactivity (green) is co-localized with the endothelial cell marker CD31 (red) both in EP1^+/+^ and EP1^-/-^ mice. B, number of CD31-positive neocortical blood vessels and % area occupied by the blood vessels do not differ between EP1^+/+^ and EP1^-/-^ mice. C, quantification of COX-1 immunoreactivity is similar between EP1^+/+^ and EP1^-/-^ mice. A.u., arbitrary unit; scale bar: 100 μm; n = 5/group.

In WT mice, neocortical apllication of the EP1 receptor inhibitor SC-51089 (1–100 μM) attenuated the increase in CBF induced by hypercapnia (pCO_2_ = 50–60 mmHg) (Fig 1A). The effect reached a plateau at a concentration of 10 μM (vehicle, 69±5%; SC-51089, 41±3%; -41±4%; p<0.05; ANOVA; Fig 1A), which was subsequently used to assess the role of EP1 receptors in the hypercapnic cerebrovascular response. In contrast, SC-51089 did not affect resting CBF or the CBF increase evoked by neocortical application of acetylcholine, the Ca^2+^ ionophore A23187 or adenosine (p>0.05; Fig 1B–1E). Similarly, SC-51089 (1–100 μM) did not affect the increase in CBF produced by whisker stimulation (p>0.05; Fig 1F). Neither EP3 (L-798,106) nor EP4 receptor (ONO-AE3-208) inhibitor, however, affected these CBF responses (p>0.05; S1 and S2 Figs). In agreement with these pharmacological data, in EP1^-/-^ mice the increase in CBF evoked by hypercapnia (pCO_2_ = 50–60 mmHg) was also attenuated (p<0.05; Fig 2A), whereas the CBF response produced by acetylcholine, A23187, adenosine, or whisker stimulation was not affected (p>0.05; Fig 2B). These effects could not be attributed to alterations in vascular structure in the null mice since the number and morphology of cerebral blood vessels in the somatosensory cortex of EP1^-/-^ did not differ from WT mice (p>0.05; Fig 3A and 3B).

### Role of COX-1-derived PGE_2_ in the increase in CBF induced by hypercapnia

**Fig 4:**
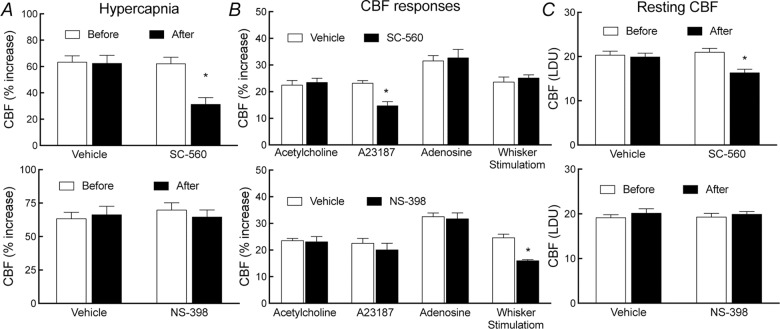
**Effect of SC-560 or NS-398 on (A) the increase in CBF elicited by hypercapnia, (B) resting CBF, or (C) CBF response to acetylcholine, A23187, adenosine, or whisker stimulation in wild-type mice**. LDU, laser-Doppler perfusion units. *p<0.05 paired t-test; n = 5/group.

The finding that PGE_2_ EP1 receptors are needed for the CBF response to hypercapnia (pCO_2_ = 50–60 mmHg) suggests the involvement of the COX-derived prostanoid PGE_2_ [11, 32]. Therefore, we next determined the source of PGE_2_: COX-1 or COX-2. In agreement with previous studies [6], neocortical superfusion of the COX-1 inhibitor SC-560 attenuated the increase in CBF evoked by hypercapnia (vehicle, 62±5%; SC-560, 31±5%; -50±5%; p<0.05; Fig 4A) or the Ca^2+^ ionophore A23187 (-37±1%; p<0.05; Fig 4B) and also reduced resting CBF (-22±1%; p<0.05; Fig 4C). However, SC-560 did not affect the increase in CBF induced by acetylcholine, adenosine, or whisker stimulation (p<0.05; Fig 4B). In contrast, the COX-2 inhibitor NS-398 did not alter resting CBF or CBF response to hypercapnia, acetylcholine, A23187 or adenosine (p>0.05; Fig 4A–4C), but it attenuated the increase in CBF induced by whisker stimulation (-35±1%; p<0.05; Fig 4B), as demonstrated previously [31]. These findings indicate that COX-1-derived PGE_2_ plays a critical role in the hypercapnic increase in CBF.

### Cellular identification of COX-1 and EP1 receptors in EP1^+/+^ and in EP1^-/-^ mice

**Fig 5:**
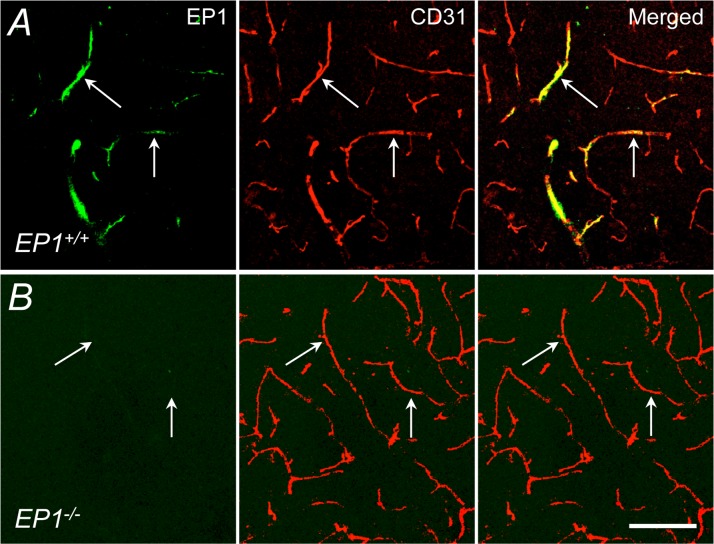
EP1 receptor immunoreactivity in CD31-positive microvessels of the somatosensory cortex. EP1 receptor immunoreactivity (green) is colocalized with CD31^+^ cortical vessels (red, arrows) in EP1^+/+^ (A), but barely detectible in EP1^-/-^ (B) mice. Scale bar, 100 μm.

In the somatosensory cortex of EP1^-/-^ and WT littermates, COX-1 immunoreactivity was localized in CD31^+^ blood vessels and Iba1^+^ microglia and its immunoreactty levels were similar (p>0.05; Fig 3A and 3C & S3 Fig). In contrast, COX-1 immunoreactivity was not observed in neurons (Neun^+^) or astrocytes (GFAP^+^) (S4A and S4B Fig). Immunoreactivity of EP1 receptors was detected in CD31^+^ cerebral microvessels and Neun^+^ neurons (Fig 5 and S5A Fig). EP1 receptor immunoreactivity was not detected in microglia (Iba1^+^) or astrocytes (GFAP^+^) (S5B and S5C Fig). Furthermore, in EP1^-/-^ mice EP1 receptor immunoreactivity was negative (Fig 5 & S5A–S5C Fig). Taken together, the data suggest that COX-1 is expressed in endothelial cells and microglia, while EP1 receptors are expressed in endothelial cells and neurons.

### Cerebrovascular responses to exogenous AA and PGE_2_ in EP1^+/+^ mice treated with SC-51089 and in EP1^-/-^ mice

PGE_2_ is synthesized from AA through enzymatic steps involving COX [11, 32]. The data presented above implicate COX-1 in the increase in CBF produced by hypercapnia. Next, we sought to determine the effect of SC-51089 (10 μM) on the CBF increase induced by neocortical application of AA (0.5–100 μM) or PGE_2_ (1–50 μM). In EP1^+/+^ mice, neocortical superfusion of AA increased CBF dose-dependently (p<0.05; Table 1). Neocortical application of PGE_2_ also increased CBF (p<0.05; Table 1), but at concentrations above 1 μM (p>0.05; Table 1). The CBF response to AA [AA (10 μM), 20±2%; AA+SC-51089, 9±1%; -55%; p<0.05] and PGE_2_ [PGE_2_ (10 μM), 16±2%; PGE_2_+SC-51089, 10±2%; -38%; p<0.05] were attenuated by neocortical application of SC-51089 (10 μM) (see Table 1). In the EP1^-/-^ mice, the CBF increase induced by AA or PGE_2_ were also attenuated, a reduction comparable to that observed in SC-51089-treated EP1^+/+^ mice (see Table 1). These findings implicate EP1 receptors in the cerebrovascular responses induced by AA or PGE_2_.

### Effect of exogenous PGE_2_ on the attenuation of the hypercapnic vasodilation by SC-560 or SC-51089

**Fig 6:**
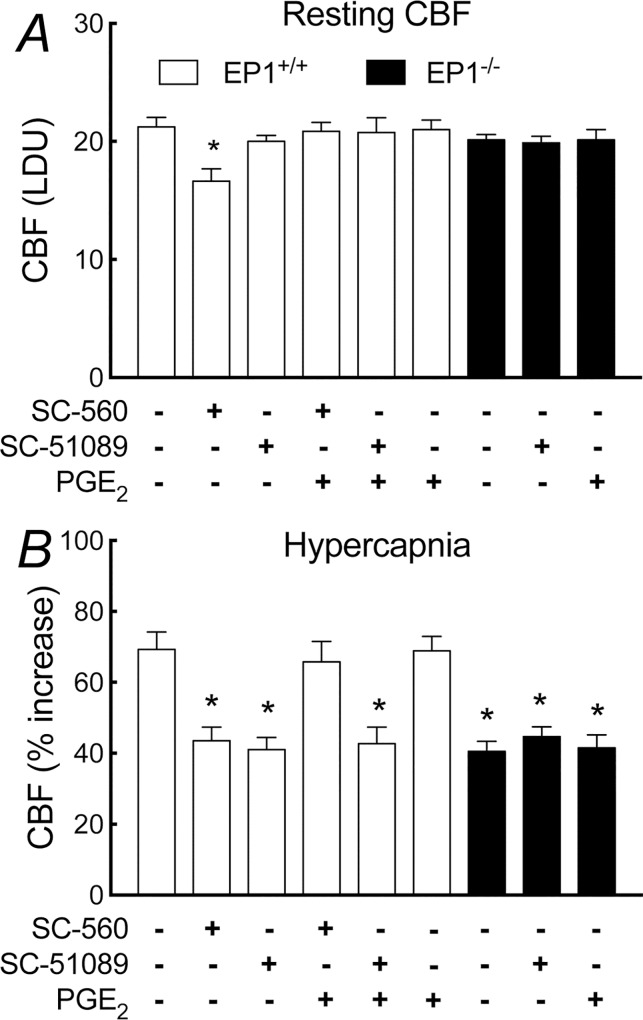
**Effect of SC-560 or SC-51089 with or without PGE**_**2**_ **on resting CBF (A) and the increase in CBF elicited by hypercapnia (B) in EP1**^**-/-**^ **and wild-type littermates**. LDU, laser-Doppler perfusion units; * p<0.05 from no treatment, analysis of variance and Tukey’s test; n = 5/group.

In these experiments, the effect of PGE_2_ on the attenuation of the hypercapnic response by SC-560 or SC-51089 was examined. On the basis of a dose response study (see Table 1), we selected a concentration of PGE_2_ (1 μM) that does not alter CBF. In WT mice, neocortical application of this concentration of PGE_2_ alone did not affect resting CBF or hypercapnic response, but completely restored the attenuation in resting CBF or hypercapnic response induced by SC-560 (25 μM) (p>0.05; Fig 6A and 6B). In contrast, PGE_2_ failed to reestablish the attenuation in the hypercapnic CBF response induced by SC-51089 (10 μM) (p<0.05; Fig 6B), indicating a role that EP1 receptors play in mediating the hypercapnic CBF effect of COX-1-derived PGE_2_ is obligatory. Consistent with these pharmacological data, PGE_2_ had no effect in EP1^-/-^ mice (Fig 6A and 6B).

## Discussion

We have demonstrated that PGE_2_ EP1 receptors are critical for the increase in CBF induced by systemic hypercapnia. Specifically, we found that SC-51089, not L-798,206 or ONO-AE3-208, attenuate the increases in CBF induced by hypercapnia, AA, or PGE_2_. In contrast, the increase in CBF induced by acetylcholine, A23187, adenosine, or whisker stimulation is not affected. These findings were further supported by corresponding observations in mice lacking EP1 receptor [15]. As shown previously, the COX-1 inhibitor SC-560 also reduced the hypercapnic response, whereas the COX-2 inhibitor NS-398 had no effect, pointing to COX-1 as a major enzyme in the effect. Accordingly, PGE_2_ counteracted the effect of SC-560 in attenuating the hypercapnic response in WT mice, attesting the participation of COX-1-derived PGE_2_. In contrast, PGE_2_ failed to counteract the effect of SC-51089, suggesting an obligatory role that EP1 receptors play in the hypercapnic responses. The morphology and number of cerebral blood vessels in the somatosensory cortex was comparable between EP1 null and WT control. Using immunofluorescent stainings to investigate the cellular locations of COX-1 and PGE_2_ EP1 receptors, we found COX-1 in endothelial cells and microgia and EP1 receptors in endothelial cells and neurons. These observations provide the first demonstration that EP1 receptors play an obligatory role in mediaiting the hypercapnic vasodilatory effect of COX-1-derived PGE_2_.

The cerebrovascular effects of pharmacological agents used can not be attributed to nonpharmacological effects causing vascular damage or to a gradual deterioration of the experimental preparation because the agents were selectively effective. For example, SC-51089 affected only the hypercapnic response or the CBF response to AA or PGE_2_, but it did not alter the CBF response to acetylcholine, A23187, adenosine, or whisker stimulation. As shown previously [6], SC-560 attenuated resting CBF or the response to hypercapnia or A23187 but not to acetyclhoiline or adenosine. Furthermore, NS-398 affected only whisker stimulation, but did not imact on other responses. Nevertheless, our findings clearly establish a role that PGE_2_ EP1 receptors play in the cerebrovascular responses induced by hypercapna.

Pharmacological inhibition or genetic ablation of EP1 receptors specifically reduces the hypercapnic response. The mechanistic pathways leading to the increase in CBF evoked by hypercapnia rely on not only vascular but also parenchymal factors [1, 32]. One of the major pathways includes the effect of acidosis on smooth muscle cells in brain blood vessels [2, 33], but many other factors derived from inside or outside of the blood vessel walls also contribute to this response. For example, a certain level of prostanoids is required for the hypercapnic response to occur [21], an effect implicating as a permissive factor but not as an active participant. Our findings that PGE_2_ does not affect resting CBF, but reestablishes the attenuation in resting CBF or hypercapnic response by SC-560 could suggest that PGE_2_ acts as one of the permissive factors. Interestingly, we found that PGE_2_ fails to reestablish the attenuation in hypercapnic vasodilation by SC-51089. This observation suggests that COX-1 is the source of prostanoids mediating resting cerebrovascular tone that does not require a role of PGE_2_ (EP1/EP3/EP4) receptors. With regard to the COX-1 by-products, PGE_2_ and prostacyclin (PGI_2_) are vasoactive [32, 34] and could be the mediator of resting CBF. However, the exact reaction products of COX-1 involved in the resting CBF remain to be determined. Taken together, the finding that the hypercapnic response is attenuated both in WT mice by acute inhibition of EP1 receptors and in EP1 null mice suggests the possibility that EP1 receptors are required for the hypercapnic response and, consequently, cannot be taken over.

The observation that EP1 receptors are required for the hypercapnic response implicates PGE_2_ in the response. PGE_2_ is a prostanoid synthesized by the COX pathway from AA. As anticipated [6], we found that SC-560, not SC-398, attenuates the increase in CBF evoked by hypercapnia, a finding indicating COX-1 as a synthesizing enzyme of PGE_2_. We found that COX-1 is localized to the wall of brain blood vessels and microglia in the somatosensory cortex, which is congruent with previous findings [14, 35]. The previous study that the effect of the nonselective COX inhibitor indomethacin on the hypercapnic increase in CBF is reversed by PGE_2_ [21] raises the possibility that this prostanoid exerts the downstream vascular effects of COX-1. Indeed, this possibility is supported by our finding that exogenous PGE_2_ reestablishes the attenuation of the hypercapnic response in WT mice induced by SC-560 but not by SC-51089. This observation indicates that in the inhibition of COX-1 PGE_2_ is able to counteract the attenuation, but in the absence of EP1 receptors it is ineffective. These findings provide additional evidence that COX-1 plays a distinct role as a sourse of PGE_2_ actuating the role of EP1 receptors in the hypercapnic vasodilation.

The present findings that PGE_2_ does not rescue the attenuation in the hypercapnic response if EP1 receptors are inhibited provide evidence that only EP1 receptors, and not other prostanoid receptors, are involved in the response. We found that EP1 receptors are localized to the endothelial cells of brain blood vessels and neurons, but not microglia, in the somatosensory cortex. However, we cannot rule out that microglial EP1 is expressed below immunocytochemical detection levels in resting state, but it becomes detectable in activated state [36, 37]. The actions of EP1 receptors are coupled to intracellular Ca^2+^ signalling [12, 15], a mechanism that is usually associated with hypertension [13, 14], ischemic brain injuries and associated blood flow responses [15, 38] or hymodynamic responses [10, 12]. Thus, the activation of EP1 receptors and its downstream pathways might determine pathophysioogical consequences. In contrast, the role of EP1 receptors in the hypercapnic response, to the best of our knowledge, has never been investigated. The observation that SC-51089 reduces the hypercapnic resonse in WT mice but does not in EP1 nulls provides a specificity for our findings that EP1 receptors are implicated in the response. We also found that EP3 or EP4 receptors are not involved in the hypercapnic CBF responses. These results are incongruent with a few studies [9, 10, 38] showing that EP1 and EP3 receptors are vasoconstrictory and EP4 receptors vasodilatory. The reasons for this disparity are not clear, although compounding effects of experimental conditions (*in vitro* vs *in vivo*), animal species (porcine vs murine), or pathophysiologcial conditions could play a role. Nonetheless, considering that EP1 receptors can increase intracellular Ca^2+^ [12, 15, 32, 38], their activation may be required to promote the intracellular Ca^2+^ necessary for activating the hypercapnic response. This possibility is supported by recent studies that Ca^2+^ dynamics contribute to vasodilation induced by PGE_2_ in the pocine cerebral arteries [12]. Taken together, our data suggest the possibility that EP1 receptor-mediated Ca^2+^ dynamics serve to “prime” the hypercapnic response by activating other enzymess, such as NOS or COX, involved in a obligatory role.

In conclusion, we have demonstrated that PGE_2_ EP1 receptors are required for the cerebrovascular response to hypercapnia, but are not involved in maintaining resting CBF or the CBF increase induced by acetylcholine, A23187, adenosine, or whisker stimulation. Consequently, EP1 null mice displayed reduced hypercapnic response similar to those observed in WT mice treated with SC-51089. In addition, findings with exogenous PGE_2_ indicated that COX-1-derived PGE_2_ is involved in the hypercapnic response. These findings indicate that the hypercapnic vasodilatation depends on COX-1-derived PGE_2_ acting on EP1 receptors and highlight the critical role that COX-1-derived PGE_2_ and EP1 receptors play in the hypercapnic response of the cerebral circulation.

## Supporting Information

S1 Fig **Effect of the EP3 receptor inhibitor L-798,106 on the increase in CBF induced by hypercapnia (A), resting CBF (B), and on the increase in CBF induced by acetylcholine, adenosine, or whisker stimulation (C) in wild-type mice**. LDU, laser-Doppler perfusion units; n = 5/group.
(TIF)

S2 Fig **Effect of the EP4 receptor inhibitor ONO-AE3-208 on the increase in CBF induced by hypercapnia (A), resting CBF (B), and on the increase in CBF induced by acetylcholine, adenosine, or whisker stimulation (C) in wild-type mice**. LDU, laser-Doppler perfusion units; n = 5/group.
(TIF)

S3 Fig COX-1 expression in the somatosensory cortex of EP1^+/+^ and EP1^-/-^ mice. COX-1 immunoreactivity (green) is co-localized with the microglial marker ionized calcium-binding adaptor molecule 1 Iba1 (green) both in EP1^+/+^ and EP1^-/-^ mice. Scale bar: 100 μm; repressenative pictures from n = 4/group.
(TIF)

S4 Fig COX-1 expression in neurons and astrocytes of the somatosensory cortex of EP1^+/+^ and EP1^-/-^ mice. COX-1 immunoreactivity (green) is not co-localized with the neuronal marker Neun (red, A) or the astrocytic marker GFAP (red, B) both in EP1^+/+^ and EP1^-/-^ mice. Scale bar: 100 μm; repressenative pictures from n = 4/group.
(TIF)

S5 Fig EP1 expression in neurons, astrocytes, and microglia of the somatosensory cortex of EP1^+/+^ and EP1^-/-^ mice. EP1 immunoreactivity (green) is co-localized with the neuronal marker Neun (red, A; arrows), but not with the astrocytic marker GFAP (red, B) or the microglial marker Iba1 (red, C) in EP1^+/+^ mice. EP1 immunoreactivity is barely detected in EP1^-/-^ mice (A-C). Scale bar: 100 μm; repressenative pictures from n = 4/group.
(TIF)

S1 Table Physiological variables for Fig 1. (DOCX)

S2 Table Physiological variables for S1 Fig. (DOCX)

S3 Table Physiological variables for S2 Fig. (DOCX)

S4 Table Physiological variables for Fig 2. (DOCX)

S5 Table Physiological variables for Fig 4. (DOCX)

S6 Table Physiological variables for Table 1. (DOCX)

S7 Table Physiological variables for Fig 5. (DOCX)
