# Supplementary figures and images for: Obligatory Role of EP1 Receptors in the Increase in Cerebral Blood Flow Produced by Hypercapnia in the Mice
Source: PLoS One. 2016 Sep 22;11(9):e0163329. doi: 10.1371/journal.pone.0163329 (PMC5033465; doi:10.1371/journal.pone.0163329)

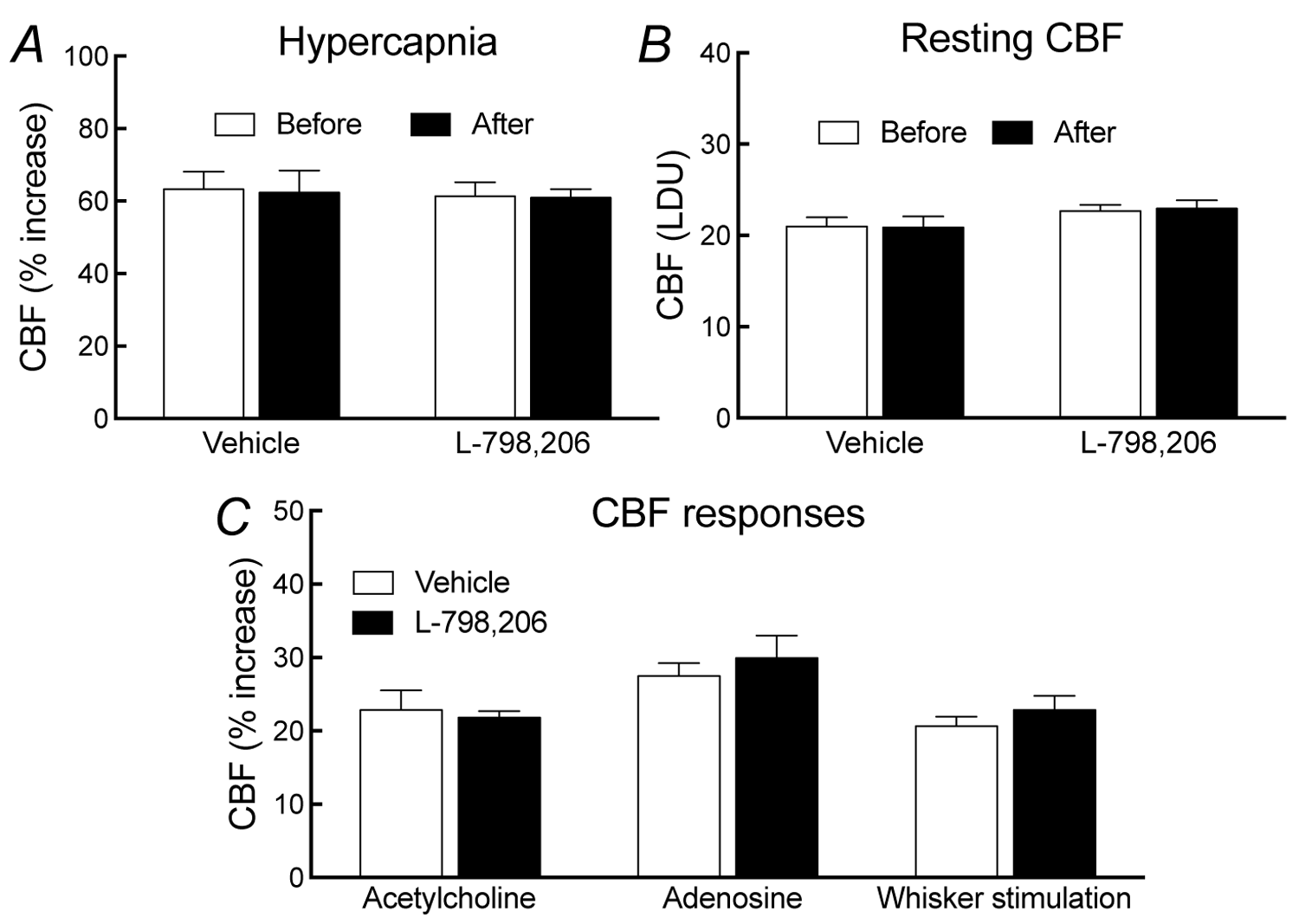

Supplement: S1 Fig — Effect of the EP3 receptor inhibitor L-798,106 on the increase in CBF induced by hypercapnia (A), resting CBF (B), and on the increase in CBF induced by acetylcholine, adenosine, or whisker stimulation (C) in wild-type mice. LDU, laser-Doppler perfusion units; n = 5/group. (TIF) [file pone.0163329.s001.tif]

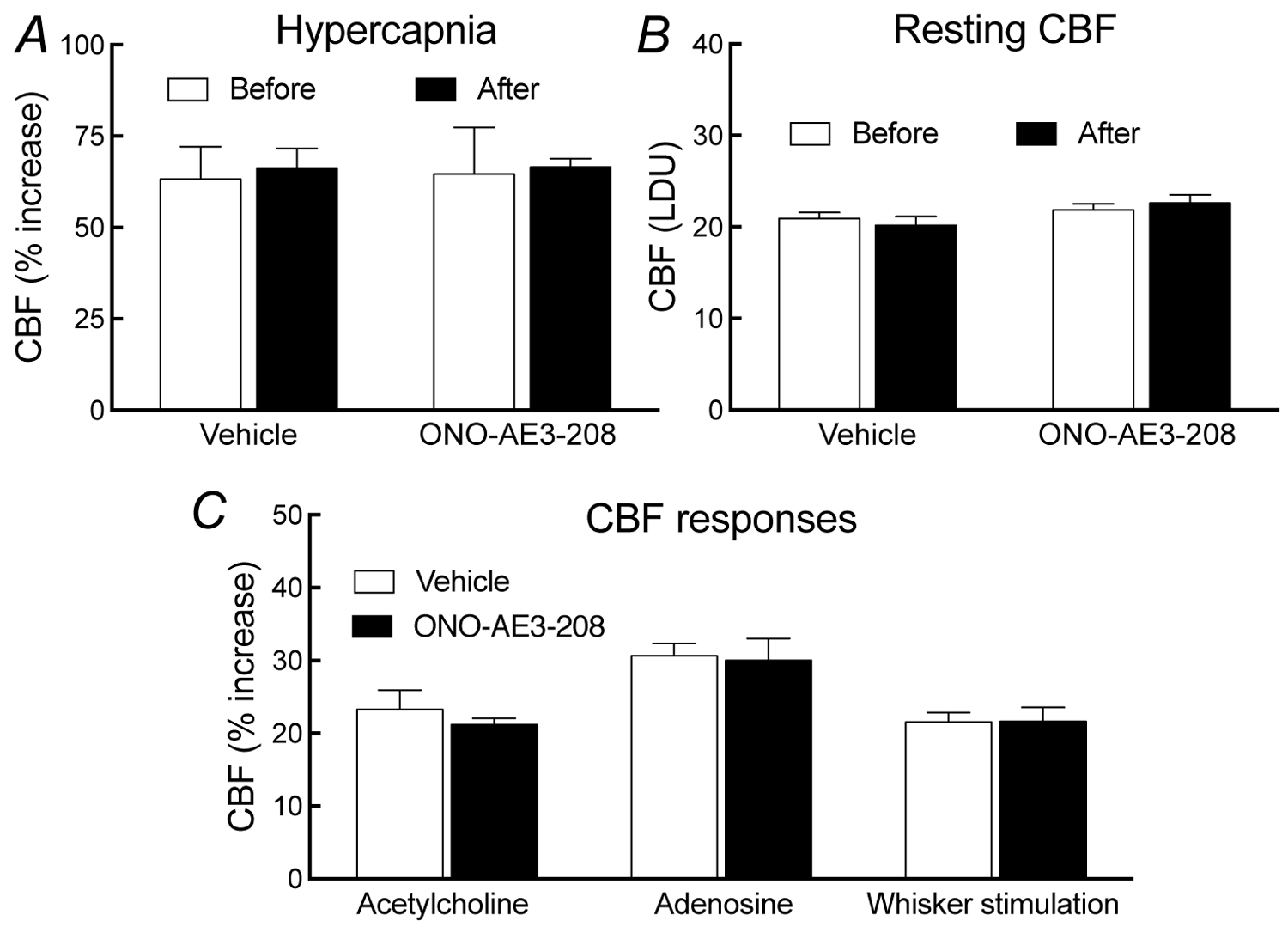

Supplement: S2 Fig — Effect of the EP4 receptor inhibitor ONO-AE3-208 on the increase in CBF induced by hypercapnia (A), resting CBF (B), and on the increase in CBF induced by acetylcholine, adenosine, or whisker stimulation (C) in wild-type mice. LDU, laser-Doppler perfusion units; n = 5/group. (TIF) [file pone.0163329.s002.tif]

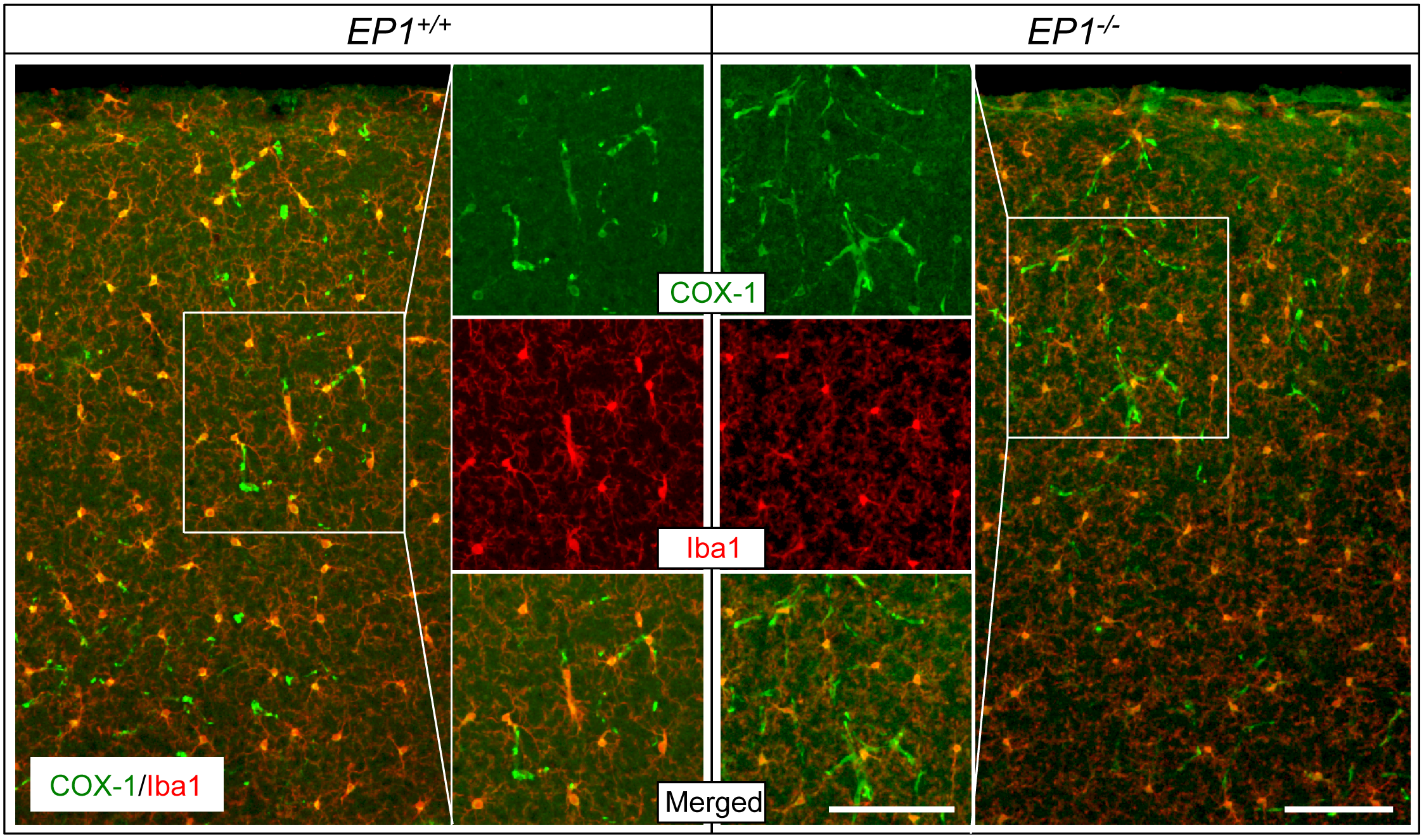

Supplement: S3 Fig — COX-1 immunoreactivity (green) is co-localized with the microglial marker ionized calcium-binding adaptor molecule 1 Iba1 (green) both in EP1+/+ and EP1-/- mice. Scale bar: 100 μm; repressenative pictures from n = 4/group. (TIF) [file pone.0163329.s003.tif]
