# Supplementary material for: Obligatory Role of EP1 Receptors in the Increase in Cerebral Blood Flow Produced by Hypercapnia in the Mice
Source: PLoS One. 2016 Sep 22;11(9):e0163329. doi: 10.1371/journal.pone.0163329 (PMC5033465; doi:10.1371/journal.pone.0163329)
Supplement: S1 Table — (DOCX) [file pone.0163329.s006.docx]

| **S1 Table. Physiological variables for Figure 1.**   \| Genotype \| Treatment \| Time \| Stimuli \| N \| MAP \| pCO_2_ \| pO_2_ \| pH \| \| --- \| --- \| --- \| --- \| --- \| --- \| --- \| --- \| --- \| \| (mmHg) \| (mmHg) \| (mmHg) \| \| WT \| SC-51089 (1 µM) \| Before \| Whisker, A23187, Adenosine \| 5 \| 83±2 \| 32.9±3.0 \| 130.1±4.0 \| 7.42±0.01 \| \| Hypercapnia \| 5 \| 83±2 \| 56.2±1.8* \| 134.2±5.8 \| 7.20±0.02* \| \| After \| Whisker, A23187, Adenosine \| 5 \| 82±4 \| 33.0±2.2 \| 130.2±6.6 \| 7.40±0.05 \| \| Hypercapnia \| 5 \| 82±2 \| 56.6±2.0* \| 133.9±8.0 \| 7.23±0.02* \| \| SC-51089 (10 µM) \| Before \| Whisker, A23187, Adenosine \| 5 \| 84±2 \| 33.1±3.4 \| 133.4±4.4 \| 7.41±0.03 \| \| Hypercapnia \| 5 \| 85±2 \| 54.4±1.8* \| 133.4±3.3 \| 7.23±0.02* \| \| After \| Whisker, A23187, Adenosine \| 5 \| 83±2 \| 31.9±2.4 \| 131.5±4.3 \| 7.40±0.01 \| \| Hypercapnia \| 5 \| 85±2 \| 53.9±1.2* \| 133.4±3.7 \| 7.23±0.02* \| \| SC-51089 (100 µM) \| Before \| Whisker, A23187, Adenosine \| 5 \| 80±2 \| 31.3±1.4 \| 136.1±6.1 \| 7.42±0.02 \| \| Hypercapnia \| 5 \| 81±4 \| 55.7±1.8* \| 132.1±6.0 \| 7.20±0.04* \| \| After \| Whisker, A23187, Adenosine \| 5 \| 80±2 \| 31.8±1.6 \| 132.3±4.9 \| 7.41±0.02 \| \| Hypercapnia \| 5 \| 80±4 \| 54.3±1.4* \| 134.8±6.9 \| 7.22±0.02* \| \| Mean±SEM; *p<0.05 vs normocapnia \| \| \| \|  \|  \|  \|  \|  \| |
| --- | --- | --- | --- | --- | --- | --- | --- | --- | --- | --- | --- | --- | --- | --- | --- | --- | --- | --- | --- | --- | --- | --- | --- | --- | --- | --- | --- | --- | --- | --- | --- | --- | --- | --- | --- | --- | --- | --- | --- | --- | --- | --- | --- | --- | --- | --- | --- | --- | --- | --- | --- | --- | --- | --- | --- | --- | --- | --- | --- | --- | --- | --- | --- | --- | --- | --- | --- | --- | --- | --- | --- | --- | --- | --- | --- | --- | --- | --- | --- | --- | --- | --- | --- | --- | --- | --- | --- | --- | --- | --- | --- | --- | --- | --- | --- | --- | --- | --- | --- | --- | --- | --- | --- |
