# Supplementary material for: Obligatory Role of EP1 Receptors in the Increase in Cerebral Blood Flow Produced by Hypercapnia in the Mice
Source: PLoS One. 2016 Sep 22;11(9):e0163329. doi: 10.1371/journal.pone.0163329 (PMC5033465; doi:10.1371/journal.pone.0163329)
Supplement: S2 Table — (DOCX) [file pone.0163329.s007.docx]

| **S2 Table. Physiological variables for S1 Figure.**   \| Genotype \| Treatment \| Time \| Stimuli \| N \| MAP \| pCO_2_ \| pO_2_ \| pH \| \| --- \| --- \| --- \| --- \| --- \| --- \| --- \| --- \| --- \| \| (mmHg) \| (mmHg) \| (mmHg) \| \| WT \| L-798,206 (1 µM) \| Before \| Whisker, \| 5 \| 80±3 \| 31.3±2.8 \| 136.9±5.0 \| 7.40±0.02 \| \| Acetylcholine, \| \| Adenosine \| \| Hypercapnia \| 5 \| 82±3 \| 56.6±1.3* \| 128.8±5.8 \| 7.20±0.01* \| \| After \| Whisker, \| 5 \| 81±3 \| 32.3±1.0 \| 126.2±2.2 \| 7.39±0.02 \| \| Acetylcholine, \| \| Adenosine \| \| Hypercapnia \| 5 \| 82±3 \| 56.2±2.5* \| 129.1±8.0 \| 7.21±0.01* \|   Mean±SEM; *p<0.05 vs normocapnia |
| --- | --- | --- | --- | --- | --- | --- | --- | --- | --- | --- | --- | --- | --- | --- | --- | --- | --- | --- | --- | --- | --- | --- | --- | --- | --- | --- | --- | --- | --- | --- | --- | --- | --- | --- | --- | --- | --- | --- | --- | --- | --- | --- | --- | --- |

| S3 Table. | | | | | | | | | |
| --- | --- | --- | --- | --- | --- | --- | --- | --- | --- |
|  | Genotype | Treatment | Time | Stimuli | N | MAP | pCO_2_ | pO_2_ | pH |
|  |  |  |  |  |  | (mmHg) | (mmHg) | (mmHg) |  |
| S2 Fig | WT | ONO-AE3-208 (1 µM) | Before | Whisker,  Acetylcholine,Adenosine | 5 | 79±6 | 34.5±2.1 | 133.9±8.0 | 7.39±0.02 |
|  |  |  |  | Hypercapnia | 5 | 83±3 | 56.8±1.6* | 128.6±9.0 | 7.19±0.02* |
|  |  |  | After | Whisker,  Acetylcholine,Adenosine | 5 | 83±7 | 30.3±1.2 | 128.2±2.3 | 7.41±0.01 |
|  |  |  |  | Hypercapnia | 5 | 85±4 | 55.2±1.0* | 130.1±6.0 | 7.10±0.02* |
| Mean±SEM; *p<0.05 vs normocapnia | | | | | | | | | |

| S4 Table | | | | | | | |
| --- | --- | --- | --- | --- | --- | --- | --- |
|  | Genotype | Stimuli | N | MAP | pCO_2_ | pO_2_ | pH |
|  |  |  |  | (mmHg) | (mmHg) | (mmHg) |  |
| Fig. 2 | EP1^+/+^ | Acetylcholine, A23187, Adenosine,  whisker stimulation | 5 | 81±2 | 35.5±2.4 | 129.0±5.4 | 7.38±0.03 |
|  |  | Hypercapnia | 5 | 81±2 | 57.6±1.7* | 131.5±5.7 | 7.18±0.02* |
|  | EP1^+/+^ | Acetylcholine, A23187, Adenosine,  whisker stimulation | 5 | 83±3 | 33.3±1.5 | 128.9±3.2 | 7.40±0.04 |
|  |  | Hypercapnia | 5 | 83±3 | 54.5±1.8* | 137.3±3.6 | 7.20±0.03* |
| Mean±SEM; *p<0.05 vs normocapnia | | | | | | | |

| S5 Table. | | | | | | | | | |
| --- | --- | --- | --- | --- | --- | --- | --- | --- | --- |
|  | Genotype | Treatment | Time | Stimuli | N | MAP | pCO_2_ | pO_2_ | pH |
|  |  |  |  |  |  | (mmHg) | (mmHg) | (mmHg) |  |
| Fig. 4 | WT | SC-560 | Before | Whisker, A23187, Adenosine | 5 | 84±3 | 32.7±2.6 | 137.6±5.6 | 7.40±0.03 |
|  |  |  |  | Hypercapnia | 5 | 84±3 | 54.6±0.9* | 131.0±5.5 | 7.24±0.03* |
|  |  |  | After | Whisker, A23187, Adenosine | 5 | 84±3 | 31.9±1.8 | 131.0±4.4 | 7.41±0.01 |
|  |  |  |  | Hypercapnia | 5 | 85±3 | 53.9±1.2* | 133.9±3.7 | 7.23±0.02* |
|  |  | NS-398 | Before | Whisker, A23187, Adenosine | 5 | 84±3 | 33.4±2.0 | 129.7±8.9 | 7.37±0.04 |
|  |  |  |  | Hypercapnia | 5 | 83±2 | 55.2±2.3* | 137.9±2.3 | 7.24±0.02* |
|  |  |  | After | Whisker, A23187, Adenosine | 5 | 84±3 | 30.1±2.8 | 131.1±9.1 | 7.35±0.01 |
|  |  |  |  | Hypercapnia | 5 | 84±2 | 54.4±2.2* | 137.9±2.3 | 7.24±0.02* |
| Mean±SEM; *p<0.05 vs normocapnia | | | | | | | | | |

| S6 Table. | | | | | | | | | |
| --- | --- | --- | --- | --- | --- | --- | --- | --- | --- |
|  | Genotype | Treatment | Stimuli | Time | N | MAP | pCO_2_ | pO_2_ | pH |
|  |  |  |  |  |  | (mmHg) | (mmHg) | (mmHg) |  |
| Table 1 | EP1^+/+^ | Vehicle | Arachidonic acid | Before | 5 | 82±1 | 31.2±2.3 | 139.8±5.7 | 7.39±0.02 |
|  |  |  |  | After | 5 | 82±2 | 28.8±1.3 | 144.8±5.8 | 7.39±0.04 |
|  |  |  | PGE2 | Before | 5 | 80±3 | 30.6±1.6 | 133.6±6.3 | 7.43±0.01 |
|  |  |  |  | After | 5 | 80±3 | 30.7±1.3 | 138.6±5.0 | 7.40±0.01 |
|  |  | SC-51089 | Arachidonic acid | Before | 5 | 83±1 | 32.0±1.7 | 137.3±6.6 | 7.39±0.02 |
|  |  |  |  | After | 5 | 83±2 | 29.1±1.3 | 143.5±2.9 | 7.39±0.04 |
|  |  |  | PGE2 | Before | 5 | 83±3 | 32.7±1.8 | 132.2±3.9 | 7.37±0.02 |
|  |  |  |  | After | 5 | 83±3 | 29.6±1.4 | 140.6±3.8 | 7.39±0.02 |
|  | EP1^-/-^ | Vehicle | Arachidonic acid | Before | 5 | 84±1 | 34.6±2.0 | 140.7±6.5 | 7.38±0.02 |
|  |  |  |  | After | 5 | 85±3 | 35.0±1.7 | 138.3±9.6 | 7.40±0.01 |
|  |  |  | PGE2 | Before | 5 | 79±3 | 37.9±1.5 | 129.3±5.7 | 7.36±0.02 |
|  |  |  |  | After | 5 | 80±2 | 37.1±1.8 | 129.6±7.5 | 7.36±0.01 |
| Mean±SEM | | | | | | | | | |

| S7 Table. | | | | | | | | |
| --- | --- | --- | --- | --- | --- | --- | --- | --- |
|  | Genotype | Treatment | Time | N | MAP | pCO_2_ | pO_2_ | pH |
|  |  |  |  |  | (mmHg) | (mmHg) | (mmHg) |  |
| Fig. 5 | EP1^+/+^ | Vehicle | Before | 5 | 82±3 | 33.4±2.9 | 128.4±4.7 | 7.38±0.02 |
|  |  |  | Hypercapnia | 5 | 85±3 | 56.8±3.4* | 136.4±6.5 | 7.17±0.02* |
|  |  |  | After | 5 | 82±2 | 33.4±2.9 | 128.4±4.6 | 7.38±0.02 |
|  |  | SC-560 | Before | 5 | 83±2 | 33.3±1.9 | 130.3±2.7 | 7.39±0.01 |
|  |  |  | Hypercapnia | 5 | 84±3 | 57.3±1.3* | 131.5±4.6 | 7.19±0.01* |
|  |  |  | After | 5 | 83±2 | 31.7±2.3 | 132.6±3.9 | 7.39±0.01 |
|  |  | SC-51089 | Before | 5 | 84±2 | 36.2±3.8 | 131.4±5.9 | 7.39±0.02 |
|  |  |  | Hypercapnia | 5 | 85±1 | 58.9±2.3* | 130.2±8.0 | 7.23±0.02* |
|  |  |  | After | 5 | 85±1 | 35.7±1.6 | 135.4±8.0 | 7.37±0.01 |
|  |  | SC-560  +PGE2 | Before | 5 | 86±2 | 32.6±2.2 | 131.5±8.2 | 7.38±0.02 |
|  |  |  | Hypercapnia | 5 | 85±3 | 57.0±1.2* | 132.8±8.1 | 7.17±0.01* |
|  |  |  | After | 5 | 84±3 | 33.0±1.8 | 132.3±8.3 | 7.38±0.01 |
|  |  | SC-51089 +PGE2 | Before | 5 | 84±2 | 38.8±2.9 | 128.6±4.6 | 7.38±0.02 |
|  |  |  | Hypercapnia | 5 | 85±2 | 58.5±2.5* | 137.7±3.3 | 7.23±0.01* |
|  |  |  | After | 5 | 84±1 | 36.4±3.5 | 129.9±8.0 | 7.39±0.01 |
|  | EP1^-/-^ | Vehicle | Before | 5 | 85±3 | 33.4±1.9 | 129.4±3.7 | 7.41±0.01 |
|  |  |  | Hypercapnia | 5 | 86±3 | 58.3±1.3* | 132.5±4.8 | 7.21±0.01* |
|  |  |  | After | 5 | 86±3 | 32.7±2.0 | 131.7±3.9 | 7.40±0.01 |
|  |  | SC-51089 | Before | 5 | 84±3 | 33.7±2.4 | 130.2±8.2 | 7.39±0.02 |
|  |  |  | Hypercapnia | 5 | 84±3 | 56.0±1.1* | 133.9±8.1 | 7.18±0.01* |
|  |  |  | After | 5 | 85±3 | 31.0±1.6 | 130.8±8.3 | 7.39±0.03 |
| Mean±SEM; *p<0.05 vs normocapnia | | | | | | | | |
