# Supplementary material for: Obligatory Role of EP1 Receptors in the Increase in Cerebral Blood Flow Produced by Hypercapnia in the Mice
Source: PLoS One. 2016 Sep 22;11(9):e0163329. doi: 10.1371/journal.pone.0163329 (PMC5033465; doi:10.1371/journal.pone.0163329)
Supplement: S3 Table — (DOCX) [file pone.0163329.s008.docx]

| **S3 Table. Physiological variables for S2 Figure.**   \| Genotype \| Treatment \| Time \| Stimuli \| N \| MAP \| pCO_2_ \| pO_2_ \| pH \| \| --- \| --- \| --- \| --- \| --- \| --- \| --- \| --- \| --- \| \| (mmHg) \| (mmHg) \| (mmHg) \| \| WT \| ONO-AE3-208 (1 µM) \| Before \| Whisker, \| 5 \| 79±6 \| 34.5±2.1 \| 133.9±8.0 \| 7.39±0.02 \| \| Acetylcholine,Adenosine \| \| Hypercapnia \| 5 \| 83±3 \| 56.8±1.6* \| 128.6±9.0 \| 7.19±0.02* \| \| After \| Whisker, \| 5 \| 83±7 \| 30.3±1.2 \| 128.2±2.3 \| 7.41±0.01 \| \| Acetylcholine,Adenosine \| \| Hypercapnia \| 5 \| 85±4 \| 55.2±1.0* \| 130.1±6.0 \| 7.10±0.02* \| |
| --- | --- | --- | --- | --- | --- | --- | --- | --- | --- | --- | --- | --- | --- | --- | --- | --- | --- | --- | --- | --- | --- | --- | --- | --- | --- | --- | --- | --- | --- | --- | --- | --- | --- | --- | --- | --- | --- | --- | --- | --- | --- | --- |

Mean±SEM; *p<0.05 vs normocapnia
