# Supplementary material for: Obligatory Role of EP1 Receptors in the Increase in Cerebral Blood Flow Produced by Hypercapnia in the Mice
Source: PLoS One. 2016 Sep 22;11(9):e0163329. doi: 10.1371/journal.pone.0163329 (PMC5033465; doi:10.1371/journal.pone.0163329)
Supplement: S4 Table — (DOCX) [file pone.0163329.s009.docx]

**S4 Table. Physiological variables for Figure 2.**

| Genotype | Stimuli | N | MAP | pCO_2_ | pO_2_ | pH |
| --- | --- | --- | --- | --- | --- | --- |
|  |  |  | (mmHg) | (mmHg) | (mmHg) |  |
| EP1^+/+^ | Acetylcholine, A23187, Adenosine, | 5 | 81±2 | 35.5±2.4 | 129.0±5.4 | 7.38±0.03 |
|  | whisker stimulation |  |  |  |  |  |
|  | Hypercapnia | 5 | 81±2 | 57.6±1.7* | 131.5±5.7 | 7.18±0.02* |
| EP1^+/+^ | Acetylcholine, A23187, Adenosine, | 5 | 83±3 | 33.3±1.5 | 128.9±3.2 | 7.40±0.04 |
|  | whisker stimulation |  |  |  |  |  |
|  | Hypercapnia | 5 | 83±3 | 54.5±1.8* | 137.3±3.6 | 7.20±0.03* |
| Mean±SEM; *p<0.05 vs normocapnia | | | | | | |
