# Supplementary material for: Obligatory Role of EP1 Receptors in the Increase in Cerebral Blood Flow Produced by Hypercapnia in the Mice
Source: PLoS One. 2016 Sep 22;11(9):e0163329. doi: 10.1371/journal.pone.0163329 (PMC5033465; doi:10.1371/journal.pone.0163329)
Supplement: S5 Table — (DOCX) [file pone.0163329.s010.docx]

**S5 Table. Physiological variables for Figure 4.**

| Genotype | Treatment | Time | Stimuli | N | MAP | pCO_2_ | pO_2_ | pH |
| --- | --- | --- | --- | --- | --- | --- | --- | --- |
|  |  |  |  |  | (mmHg) | (mmHg) | (mmHg) |  |
| WT | SC-560 | Before | Whisker, A23187, Adenosine | 5 | 84±3 | 32.7±2.6 | 137.6±5.6 | 7.40±0.03 |
|  |  |  | Hypercapnia | 5 | 84±3 | 54.6±0.9* | 131.0±5.5 | 7.24±0.03* |
|  |  | After | Whisker, A23187, Adenosine | 5 | 84±3 | 31.9±1.8 | 131.0±4.4 | 7.41±0.01 |
|  |  |  | Hypercapnia | 5 | 85±3 | 53.9±1.2* | 133.9±3.7 | 7.23±0.02* |
|  | NS-398 | Before | Whisker, A23187, Adenosine | 5 | 84±3 | 33.4±2.0 | 129.7±8.9 | 7.37±0.04 |
|  |  |  | Hypercapnia | 5 | 83±2 | 55.2±2.3* | 137.9±2.3 | 7.24±0.02* |
|  |  | After | Whisker, A23187, Adenosine | 5 | 84±3 | 30.1±2.8 | 131.1±9.1 | 7.35±0.01 |
|  |  |  | Hypercapnia | 5 | 84±2 | 54.4±2.2* | 137.9±2.3 | 7.24±0.02* |

Mean±SEM; *p<0.05 vs normocapnia
