# Supplementary material for: Obligatory Role of EP1 Receptors in the Increase in Cerebral Blood Flow Produced by Hypercapnia in the Mice
Source: PLoS One. 2016 Sep 22;11(9):e0163329. doi: 10.1371/journal.pone.0163329 (PMC5033465; doi:10.1371/journal.pone.0163329)
Supplement: S6 Table — (DOCX) [file pone.0163329.s011.docx]

**S6 Table. Physiological variables for Table 1.**

|  |  | Stimuli | Time | N | MAP | pCO_2_ | pO_2_ | pH |
| --- | --- | --- | --- | --- | --- | --- | --- | --- |
| Genotype | Treatment |  |  |  | (mmHg) | (mmHg) | (mmHg) |  |
| EP1^+/+^ | Vehicle | Arachidonic acid | Before | 5 | 82±1 | 31.2±2.3 | 139.8±5.7 | 7.39±0.02 |
|  |  |  | After | 5 | 82±2 | 28.8±1.3 | 144.8±5.8 | 7.39±0.04 |
|  |  | PGE2 | Before | 5 | 80±3 | 30.6±1.6 | 133.6±6.3 | 7.43±0.01 |
|  |  |  | After | 5 | 80±3 | 30.7±1.3 | 138.6±5.0 | 7.40±0.01 |
|  | SC-51089 | Arachidonic acid | Before | 5 | 83±1 | 32.0±1.7 | 137.3±6.6 | 7.39±0.02 |
|  |  |  | After | 5 | 83±2 | 29.1±1.3 | 143.5±2.9 | 7.39±0.04 |
|  |  | PGE2 | Before | 5 | 83±3 | 32.7±1.8 | 132.2±3.9 | 7.37±0.02 |
|  |  |  | After | 5 | 83±3 | 29.6±1.4 | 140.6±3.8 | 7.39±0.02 |
| EP1^-/-^ | Vehicle | Arachidonic acid | Before | 5 | 84±1 | 34.6±2.0 | 140.7±6.5 | 7.38±0.02 |
|  |  |  | After | 5 | 85±3 | 35.0±1.7 | 138.3±9.6 | 7.40±0.01 |
|  |  | PGE2 | Before | 5 | 79±3 | 37.9±1.5 | 129.3±5.7 | 7.36±0.02 |
|  |  |  | After | 5 | 80±2 | 37.1±1.8 | 129.6±7.5 | 7.36±0.01 |

Mean±SEM
