# Supplementary material for: Obligatory Role of EP1 Receptors in the Increase in Cerebral Blood Flow Produced by Hypercapnia in the Mice
Source: PLoS One. 2016 Sep 22;11(9):e0163329. doi: 10.1371/journal.pone.0163329 (PMC5033465; doi:10.1371/journal.pone.0163329)
Supplement: S7 Table — (DOCX) [file pone.0163329.s012.docx]

**S7 Table. Physiological variables for Figure 5.**

| Genotype | Treatment | Time | N | MAP | pCO_2_ | pO_2_ | pH |
| --- | --- | --- | --- | --- | --- | --- | --- |
|  |  |  |  | (mmHg) | (mmHg) | (mmHg) |  |
| EP1^+/+^ | Vehicle | Before | 5 | 82±3 | 33.4±2.9 | 128.4±4.7 | 7.38±0.02 |
|  |  | Hypercapnia | 5 | 85±3 | 56.8±3.4* | 136.4±6.5 | 7.17±0.02* |
|  |  | After | 5 | 82±2 | 33.4±2.9 | 128.4±4.6 | 7.38±0.02 |
|  | SC-560 | Before | 5 | 83±2 | 33.3±1.9 | 130.3±2.7 | 7.39±0.01 |
|  |  | Hypercapnia | 5 | 84±3 | 57.3±1.3* | 131.5±4.6 | 7.19±0.01* |
|  |  | After | 5 | 83±2 | 31.7±2.3 | 132.6±3.9 | 7.39±0.01 |
|  | SC-51089 | Before | 5 | 84±2 | 36.2±3.8 | 131.4±5.9 | 7.39±0.02 |
|  |  | Hypercapnia | 5 | 85±1 | 58.9±2.3* | 130.2±8.0 | 7.23±0.02* |
|  |  | After | 5 | 85±1 | 35.7±1.6 | 135.4±8.0 | 7.37±0.01 |
|  | SC-560 | Before | 5 | 86±2 | 32.6±2.2 | 131.5±8.2 | 7.38±0.02 |
|  |  | Hypercapnia | 5 | 85±3 | 57.0±1.2* | 132.8±8.1 | 7.17±0.01* |
|  |  | After | 5 | 84±3 | 33.0±1.8 | 132.3±8.3 | 7.38±0.01 |
|  | SC-51089 +PGE2 | Before | 5 | 84±2 | 38.8±2.9 | 128.6±4.6 | 7.38±0.02 |
|  |  | Hypercapnia | 5 | 85±2 | 58.5±2.5* | 137.7±3.3 | 7.23±0.01* |
|  |  | After | 5 | 84±1 | 36.4±3.5 | 129.9±8.0 | 7.39±0.01 |
| EP1^-/-^ | Vehicle | Before | 5 | 85±3 | 33.4±1.9 | 129.4±3.7 | 7.41±0.01 |
|  |  | Hypercapnia | 5 | 86±3 | 58.3±1.3* | 132.5±4.8 | 7.21±0.01* |
|  |  | After | 5 | 86±3 | 32.7±2.0 | 131.7±3.9 | 7.40±0.01 |
|  | SC-51089 | Before | 5 | 84±3 | 33.7±2.4 | 130.2±8.2 | 7.39±0.02 |
|  |  | Hypercapnia | 5 | 84±3 | 56.0±1.1* | 133.9±8.1 | 7.18±0.01* |
|  |  | After | 5 | 85±3 | 31.0±1.6 | 130.8±8.3 | 7.39±0.03 |

Mean±SEM; *p<0.05 vs normocapnia
